# Differences in Characteristics of Trauma Between General Trauma and Suicidal Trauma–Trauma Mechanism, Injury Site, and Severity: A Single-Center Study

**DOI:** 10.1155/emmi/6058288

**Published:** 2025-05-23

**Authors:** Ji Young Hyun, Yae Jun Son, SuHyun Kim, Keum Seok Bae, Jae Sik Chung, Il Hwan Park, Young Un Choi

**Affiliations:** ^1^CHA University School of Medicine, Seongnam 24341, Republic of Korea; ^2^Department of Medical Informatics and Biostatistics, Artificial Intelligence Big Data Medical Center, Yonsei University Wonju College of Medicine, Wonju 26426, Republic of Korea; ^3^Trauma Center, Wonju Severance Christian Hospital, Wonju 26426, Republic of Korea; ^4^Department of Surgery, Yonsei University Wonju College of Medicine, Wonju 26426, Republic of Korea; ^5^Department of Thoracic Surgery, Yonsei University Wonju College of Medicine, Wonju 26426, Republic of Korea

**Keywords:** abbreviated injury scale, injury severity score, self-harm, trauma mechanism

## Abstract

**Background:** Among patients with trauma, those with self-harm exhibit different characteristics than those who experience general accidents. Unstable vital signs following a severe injury often limit accurate imaging and injury assessment during initial treatment, rendering decision-making challenging for definitive care. Identifying correlations between damage area and severity can improve predictions and treatment decisions. We identify differences in characteristics of trauma between patients who experienced general accidents and those who attempted suicide.

**Methods:** This study investigates differences in trauma characteristics between accidental and self-harm injuries in a single-center cohort of 10,180 patients (2015–2023). We analyzed age, sex, trauma mechanism, intention of suicide, Abbreviated Injury Scale (AIS) score, injury severity score (ISS), and height for falls. We divided intentionality into accident and self-harm and analyzed and compared their characteristics.

**Results:** The self-harm group was significantly younger (mean: 10 years younger, *p* < 0.05) and had a higher proportion of falls (41.0%) and stab injuries (48.9%). Self-harm falls were more severe, with a mean fall height of 10.8m (vs. 4.14m in the accidental group, *p* < 0.001), resulting in a higher ISS (18.6 vs. 15.5, *p* < 0.05). In the self-harm group, fall attempts were common among teenagers and those in their 20s, while knife injuries were common in individuals in their 40s and 50s.

**Conclusion:** This study confirmed the relationship between the injury mechanism and AIS in patients with trauma. The damaged area and degree of damage differed between the self-harm and accident groups, even with the same mechanism. Understanding these patterns can enhance initial ER assessments and reduce missed injuries in high-risk patients.

## 1. Introduction

According to the Korea National Statistical Office's “2022 Cause of Death Statistics Results,” the proportion of deaths due to external causes other than disease among all deaths was 7.2%, and the order of deaths was suicide (25.2 per 100,000 people), transportation accidents (6.8 per 100,000 people), and falls (5.2 per 100,000 people). Suicide was the sixth leading cause of death overall, ranking first among those in their 10s, 20s, and 30s, second among those in their 50s, and fifth among those in their 60s [1]. The main suicide methods were hanging, falling, and gas poisoning from 2016 to 2020, except in 2019 [2].

Notably, most suicide attempts are unsuccessful. Therefore, the proportion of patients who attempted suicide and were transferred to the emergency room is increasing, and it is necessary to identify the characteristics of patients who attempted suicide and include them in the initial treatment strategy.

In addition, when treating patients with trauma, accurate identification of the damaged area is important for providing appropriate initial assessment. To enhance the accuracy of this identification, imaging tests, including computed tomography (CT) as an adjunctive secondary survey, are performed in addition to physical examinations [3]. However, in severely injured patients with unstable vital signs or unclear consciousness, performing imaging tests and identifying the damaged area can be challenging, potentially resulting in missed injuries [3, 4]. Such missed injuries occur in approximately 38% of secondary surveys [5] and can be avoided by repeatedly evaluating the suspected damaged areas [3, 6]. A previous study confirmed the correlation between injury mechanisms and damaged areas in patients with trauma [7]. In our hospital, we considered using this correlation for initial treatment in cases when an adjunctive secondary survey was not feasible. However, because people who attempt suicide do not intentionally wear safety equipment or take protective measures, the pattern of damaged areas, according to the mechanism of injury, is presumed to differ from that of patients with general injuries. In addition, differences in the trauma mechanism, frequency, and damaged area are expected compared to those in patients with general accidents. However, existing studies on trauma severity rarely compare self-harm mechanisms to accidental trauma. This study aims to fill this gap by analyzing trauma patterns in self-harm cases.

According to the 2022 injury type and cause statistics, unlike patients who attempted suicide, 73% of patients injured in general transportation accidents wore seatbelts, and the number of motorcycle and bicycle riders wearing helmets is increasing annually [8]. Considering that people who attempt suicide in road accidents have a lower frequency but a higher mortality rate than those with general unintentional injuries [8], it is expected that even if the same injury mechanism occurs, the damaged area may appear differently based on patient characteristics. We hypothesize that self-harm injuries, particularly falls, will have distinct severity patterns compared to accidental trauma due to differences in protective mechanisms and impact absorption.

## 2. Methods

### 2.1. Ethical Approval

This study was performed in accordance with the ethical standards as laid down in the 1964 Declaration of Helsinki and its later amendments or comparable ethical standards and was approved by Wonju Severance Christian Hospital institutional review board (IRB No. CR 323174). Informed consent was waived because of the retrospective nature of the study and the use of anonymized clinical data for analysis.

### 2.2. Study Details—Patient Enrollment and Data Collection

We retrospectively reviewed the data of patients with trauma transferred to the emergency room of our hospital from January 1, 2015, to December 31, 2023, excluding patients who died upon arrival, pregnant women, patients with no medical records, and patients who objected to treatment. Age, sex, trauma mechanism, suicide attempt, Abbreviated Injury Scale (AIS) score, injury severity score (ISS), and fall height were analyzed in 10,180 patients. Fall height was estimated using witness reports, scene assessment, and emergency responder documentation. Trauma mechanisms were categorized into traffic accidents, falls, crushes, slipping, stab or cut wounds, machines, others (sexual assault, chemical burn, and electric shock), choking, nonchemical burn, drowning, and unknown. Intentionality was categorized into accidents (9,715), self-harm (307), and assault by others (158). Patients who attempted hanging without cervical cord injury, pregnant women, patients under 15 years of age, patients with medical conditions such as poisoning drug abuse, and assault patients were excluded. Only hanging patients with cervical cord injuries were treated by the trauma team, and patients without cervical cord injuries could not be identified. Patients under 15 were excluded due to different physiological trauma responses and ethical concerns in retrospective studies. Using these data, the accident and self-harm groups were separated, and the characteristics of the two groups were analyzed and compared.

### 2.3. Inclusion Criteria

- Trauma patients transferred to the emergency room (January 2015–December 2023): 10,180 patients

### 2.4. Exclusion Criteria

- Hanging without surgical treatment
- Poisoning
- Drug abuse
- Assault
- Pregnant women
- Age under 15 years

### 2.5. Statistical Analysis

Categorical variables are expressed as frequencies and percentages. Normality and homogeneity of variance tests (Levene's test) were conducted for group comparisons. The Kruskal–Wallis test was used to analyze the differences in AIS scores among injury sites within the same injury mechanism. Post hoc tests between the AIS scores of the two injury sites were performed using the Dunn procedure, with *p* values adjusted using the Bonferroni correction. The *t*-test was used to compare AIS scores for different injury sites between the general accidental and self-harm groups in the fall injury mechanism. Tests for normality and homogeneity of variances and *t*-tests were performed using SAS software (Version 9.4; SAS Institute Inc., Cary, NC, The United States). Dunn procedure was performed using R software Version 4.3.1. The Kruskal–Wallis test was cross-validated using both SAS 9.4 and R 4.3.1, and statistical significance was set at *p* < 0.05.

## 3. Results

**Figure 1:**
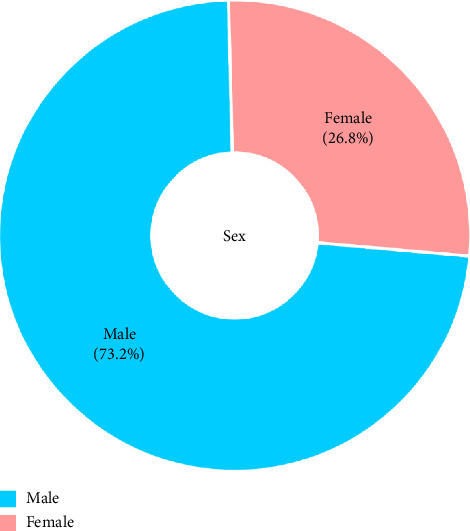
Sex distribution of all patients.

**Figure 2:**
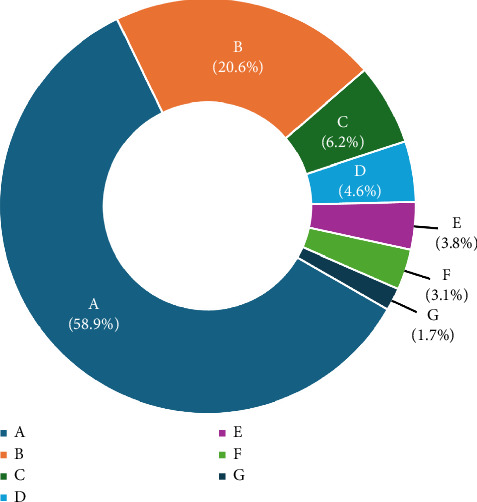
The percentage of patients who experienced each type of trauma mechanism. A: traffic accident; B: fall; C: crush; D: slipping; E: stab or cutting; F: unknown; G: machine. trauma mechanisms with a frequency below 1% were excluded from the figure.

Among the 10,180 patients, 7452 (73.2%) were men, and the most common trauma mechanisms were traffic accidents (58.9%), falls (20.6%), crushes (6.2%), slipping (4.6%), and stab or cut wounds (3.8%) (Table 1, Figures 1 and 2).

**Figure 3:**
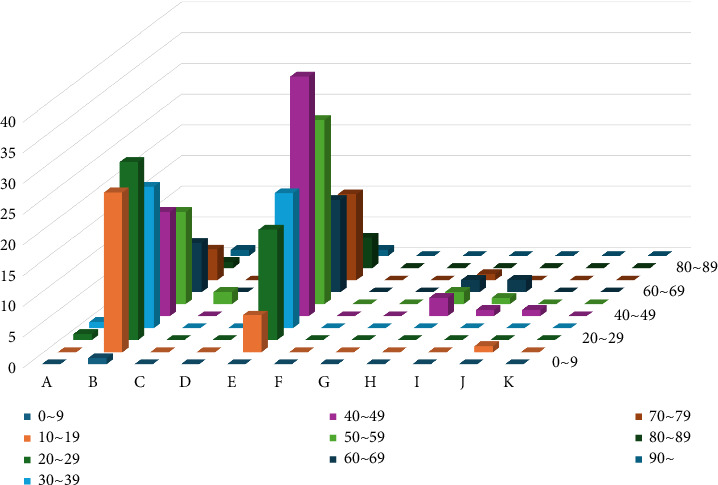
Number of patients in the self-harm group, categorized by age, who experienced each type of trauma mechanism. A: traffic accident; B: fall; C: crush; D: slipping; E: stab or cutting; F: machine; G: others (sexual assault, chemical burn, electric shock); H: choking; I: nonchemical burn; J: drowning; K: unknown.

Age and trauma mechanisms differed between the accident and self-harm groups. In the self-harm group, the frequencies of falls (41.0%) and stab or cut wounds (48.9%) were notably high, and the average age was 10 years younger than that of the accident group. Among the 1967 patients in the accidental fall group, the fall height of 777 people was confirmed, with an average of 4.14 m, and an ISS of 15.5 points. The fall height of 57 of the 126 patients in the self-harm group was confirmed, with an average of 10.8 m, and ISS of 18.6 points (Table 2 and Figure 3).

Confirming the trauma mechanism and ISS of all patients revealed significant and independent differences in ISS scores for each trauma mechanism. This result indicates differences in the damaged area for each mechanism (Table 3), suggesting that injury site and severity are determined by each mechanism.

When comparing the trauma mechanism and ISS of only severely injured patients (ISS ≥ 16 —This was chosen as it indicates major trauma requiring intensive care interventions, aligning with prior trauma severity classification standards) in the accident and self-harm groups, all damaged areas for each accident mechanism exhibited independent and significant differences in scores within the accident group (Table 4). In the self-harm group, the mechanisms showing normality and statistical significance decreased.

When the self-harm group was divided by age and mechanism, the main injury mechanism (fall) occurred in those aged 10–60 years, with a high proportion of patients in their 10s–20s (43.6%). Stab and cut wounds were more common among those aged 20 and 70 years, with a prevalence of 46% in individuals in their 40s and 50s (Table 5 and Figure 3). The higher incidence of self-inflicted stab wounds in the 40s–50s group may reflect underlying psychiatric conditions such as major depressive disorder or psychosis, which have higher prevalence in this age bracket.

Table 6 compares the AIS scores for different injury sites between general accident group and self-harm group, specifically for the fall-down injury mechanism. The effect size was calculated using the formula $r=\left(Z/\sqrt{N}\right)$. AIS1 and AIS 4 were found to be statistically significant, and AIS5 showed a markably significant statistical difference. The effect size *r* for AIS5 was 0.14, small effect, but clinically significant, indicating that the self-harm group has slightly more severe injuries compared to accidental group.

Comparing the effect size of only falls between the accident and self-harm groups revealed a higher severity of damage to the pelvis and extremities than that in the accident group.

## 4. Discussion

The analysis of trauma characteristics revealed significant differences between patients with accidental injuries and those with self-harm injuries. Notably, self-harm patients experienced higher ISS compared to accidental injury patients, particularly in falls, where the average fall height and ISS were markedly greater in the self-harm group.

In the entire trauma group, the AIS according to the injury mechanism was significantly different.

Typically, when the effect size *r* is 0.1, it means a small effect, when 0.3, it means a medium effect, and when 0.5, it means a large effect. For AIS 5, the effect size falls into the small effect category but is intermediate or beyond the point of effect, and the difference is the most prominent when compared to other damaged area. This allows us to conclude that the AIS 5 degree of damage in the self-harm group is statistically significantly greater than accident group. In particular, ISS scores of 16 or higher are considered as severe trauma patients, and this was confirmed to be 18.6 in the accident group and 15.5 in the suicide group, so the pelvic and limb damage indicated by AIS 5 was a clear difference.

Thus, when it is difficult to confirm the exact damaged area through imaging examination during the initial treatment, the injury mechanism can be useful for predicting the damaged area. Unlike the general accident group, which followed the characteristics of the entire trauma group, the self-harm group had an overwhelming number of cases of falling and self-injury using knives. While the injury severity was high in falls, it was relatively low for stab and cut wounds. Specifically, among falls, the damage to the pelvis and extremities was more severe in the self-harm group than in the accident group. Unlike stabbing or cutting, where the damaged area was relatively clear, it was difficult to determine the damaged area in falls without imaging examination during initial treatment. Therefore, predicting the damaged areas in cases where imaging examinations are limited is crucial. When a patient with fall injuries, suspected of attempting suicide, is transported to the emergency room, it is anticipated that the fall height will be higher, the ISS will be greater, and the damage to the pelvis and extremities will be more severe compared to those in cases of general accidents. Given the higher severity of self-harm falls, trauma teams should prioritize whole-body CT imaging for these patients, even when external injuries appear minor.

Falling is a common method of suicide attempt in many countries. However, there are only a few studies on the damage area caused by falls. People who attempt suicide tend to have “feet-first landings,” resulting in more fractures of the pelvis and lower extremities, and fewer skull fractures as the lower extremities absorb shock [9]. However, another study indicated that while people who attempt suicide tends to exhibit “feet-first landings,” they also experience many injuries to the face, abdomen, and extremities [10]. These studies have limitations in that the number of suicide attempts was relatively small at 57 and 40, respectively, and the height of the fall was not presented. In the present study, we confirmed that the average fall height of those who attempted suicide was 10.8 m, which is more than twice the height of accidental falls, and the damage to the pelvis and limbs was more severe than that in the accident group. Nevertheless, additional research is required to examine the relationship between falls and damaged areas.

In the present study, the average age of the self-harm group was approximately 10 years younger than that of the accident group, showing similar results to Korean statistics, where the age of people attempting suicide is decreasing [11]. Moreover, falls were more common among teenagers and patients in their 20s, while stabbing or cutting was more common among people in their 40s and 50s. The average age of those who attempted suicide by falling was the same as that reported in a previous study in Korea [12]. Although various factors may influence this trend, the decrease in age could be attributed to media influence and the prevalence of easily accessible high-rise buildings in Korean cities [13]. According to a previous study in the United Kingdom, self-harm using knives was the most common among people aged 25–54 [14]. The distinct trauma patterns in self-harm cases may be linked to psychiatric diagnoses, necessitating routine psychiatric screening in ER settings. According to a previous study in Korea, depressive disorder was more common among patients who opted for lethal methods of suicide, such as falling, inhaling pesticides, briquettes, and hanging, than in those who chose less lethal methods, such as suicide using a knife or drug addiction. Specifically, schizophrenia and adjustment disorders were more prevalent among patients who chose lethal suicide methods [15]. As the present study did not include psychiatric diagnostic data according to age and injury mechanism, future research is required to determine whether there is an association across specific age groups, psychiatric diagnoses, and suicide mechanisms.

Despite the long-standing interest in preventing suicide, Korea's suicide rate remains the highest among OECD countries [16], and in 2022, self-harm/suicide was the leading cause of death, excluding diseases [2]. According to a survey on the reasons for suicide attempts in 2022, psychiatric problems were the most common at 44% [8]. This demonstrates the importance of accurate psychiatric diagnosis and continuous follow-up to reduce suicide rates. Future research is needed to investigate the rate at which patients admitted to the emergency room for a suicide attempt receive a psychiatric diagnosis through psychiatric treatment. Moreover, studies should examine the relationship between the extent of psychiatric treatment and the frequency of repeated suicide attempts.

This study had some limitations. First, general statistical analysis could be applied in the general accident group because the number of patients was sufficiently large, and the data followed a normal distribution. However, the self-harm group data did not follow a normal distribution owing to the small number of patients, except for those with falls. Therefore, various statistical methods have been proposed to address this issue. Second, patients who died following a suicide attempt were excluded from the study, which limits the representation characteristics of all patients who attempt suicide. Third, suicide attempts other than trauma, such as CO inhalation, pesticide use, and hanging, were excluded from this study; therefore, patients with nontraumatic suicide attempts were not identified. Fourth, the purpose of this study was to identify the initial degree of injury and the characteristics of the injured area in trauma patients; thus, it cannot show the mortality or morbidity of the patients. Fifth, fall height was estimated using witness reports, scene assessment, and emergency responder documentation. However, there were cases where the patient was found fallen without a witness or in a place other than the residence, making it difficult to confirm the exact height. Therefore, in the case of the accident group, only 777 out of 1968 people were confirmed, and in the suicide group only 57 out of 126 people were confirmed. Although the data are representative to the greatest extent possible, it cannot be said to represent the exact average of all groups. Sixth, as this study did not include a psychiatric analysis, additional subgroup analyses of psychiatric diagnoses and mechanisms in the suicide group are needed. Finally, this was a single-center study, and the geographic and age characteristics of the region where the institute was located were different from those of other regions. In particular, it may be difficult to generalize the research results because the average age in the region covered by this organization is higher than that in other regions, and the population density is low.

## 5. Conclusions

We confirmed the relationship between the injury mechanism and AIS in patients with trauma and compared the self-harm and accident groups, confirming that the damaged area and degree of damage differ, even if the mechanism is the same. In the self-harm group, falls and self-injury with knives were common, and falls were characteristically different from those in the general accident group in terms of height, severity, and area of damage. Emergency physicians should be aware that self-harm falls result in significantly greater injury severity, necessitating aggressive early assessment and monitoring. This study highlights the potential of predicting the damaged area based on the mechanism of injury, providing important insights for early diagnosis and treatment. Moreover, future studies should explore psychiatric diagnoses in trauma patients to refine risk prediction models.

## Figures and Tables

**Table 1:** All patients' clinical characteristics analyzed by mechanisms.

	Overall^1^ (*N* = 10,180)	A^1^ (*N* = 5992)	B^1^ (*N* = 2098)	C^1^ (*N* = 634)	D^1^ (*N* = 473)	E^1^ (*N* = 385)	F^1^ (*N* = 177)	G^1^ (*N* = 84)	H^1^ (*N* = 9)	I^1^ (*N* = 7)	J^1^ (*N* = 5)	K^1^ (*N* = 316)
Age (years)	53.4 ± 19.5	52.0 ± 20.4	54.6 ± 17.8	54.5 ± 17.4	64.5 ± 18.4	48.0 ± 16.9	55.8 ± 14.0	50.0 ± 16.4	57.0 ± 12.1	47.6 ± 18.8	33.4 ± 12.0	61.8 ± 16.8
Sex												
Female	2728 (26.8)	1804 (30.1)	440 (21.0)	106 (16.7)	167 (35.3)	103 (26.8)	14 (7.9)	15 (17.9)	2 (22.2)	2 (28.6)	2 (40.0)	73 (23.1)
Male	7452 (73.2)	4188 (69.9)	1658 (79.0)	528 (83.3)	306 (64.7)	282 (73.2)	163 (92.1)	69 (82.1)	7 (77.8)	5 (71.4)	3 (60.0)	243 (76.9)
ISS	13.5 ± 10.2	12.9 ± 10.2	15.7 ± 10.3	14.1 ± 8.7	14.2 ± 9.3	6.7 ± 7.5	10.9 ± 9.4	16.0 ± 15.9	14.1 ± 10.5	17.9 ± 19.5	11.4 ± 9.0	18.6 ± 9.8
ISS ≥ 16	3895 (38.3)	2060 (34.4)	706 (33.7)	270 (42.6)	219 (46.3)	43 (11.2)	42 (23.7)	40 (47.6)	5 (55.6)	3 (42.9)	2 (40.0)	210 (66.5)
AIS1	2.8 ± 1.4	2.5 ± 1.3	3.0 ± 1.4	3.2 ± 1.4	3.6 ± 1.2	2.0 ± 1.4	2.9 ± 1.8	2.5 ± 1.1	4.0 ± 1.1	2.0 ± 0.0	3.3 ± 1.5	3.8 ± 1.3
AIS1 ≥ 3	2721 (26.7)	1284 (21.4)	706 (33.7)	202 (31.9)	259 (54.8)	26 (6.8)	17 (9.6)	4 (4.8)	8 (88.9)	0 (0.0)	2 (40.0)	213 (67.4)
AIS2	1.4 ± 0.6	1.4 ± 0.6	1.5 ± 0.6	1.4 ± 0.6	1.4 ± 0.5	1.3 ± 0.5	1.6 ± 0.6	1.3 ± 0.5	0.0 ± 0.0	1.0 ± 0.0	0.0 ± 0.0	1.5 ± 0.6
AIS2 ≥ 3	104 (1.0)	72 (1.2)	20 (1.0)	7 (1.1)	0 (0.0)	1 (0.3)	1 (0.6)	0 (0.0)	0 (0.0)	0 (0.0)	0 (0.0)	3 (0.9)
AIS3	2.7 ± 1.2	2.6 ± 1.2	2.9 ± 1.2	2.7 ± 0.9	2.9 ± 1.1	2.4 ± 1.2	2.6 ± 0.7	2.6 ± 1.0	2.5 ± 0.7	3.0 ± 1.4	2.0 ± 0.0	2.8 ± 1.4
AIS3 ≥ 3	2687 (26.4)	1635 (27.3)	677 (32.3)	164 (25.9)	57 (12.1)	51 (13.2)	32 (18.1)	5 (6.0)	1 (11.1)	1 (14.3)	0 (0.0)	64 (20.3)
AIS4	2.3 ± 1.0	2.3 ± 1.1	2.4 ± 0.8	2.5 ± 0.7	2.5 ± 0.7	1.9 ± 1.1	2.5 ± 1.1	2.2 ± 0.4	2.0 ± 0.0	4.0 ± 0.0	0.0 ± 0.0	2.3 ± 0.8
AIS4 ≥ 3	1059 (10.4)	625 (10.4)	240 (11.4)	83 (13.1)	31 (6.6)	52 (13.5)	12 (6.8)	1 (1.2)	0 (0.0)	1 (14.3)	0 (0.0)	14 (4.4)
AIS5	2.1 ± 0.9	2.0 ± 0.9	2.2 ± 0.9	2.3 ± 1.0	2.1 ± 0.8	1.7 ± 0.8	2.4 ± 0.9	2.1 ± 0.9	1.5 ± 0.7	2.0 ± 0.0	0.0 ± 0.0	2.0 ± 1.1
AIS5 ≥ 3	1390 (13.7)	852 (14.2)	308 (14.7)	91 (14.4)	28 (5.9)	25 (6.5)	56 (31.6)	4 (4.8)	0 (0.0)	0 (0.0)	0 (0.0)	26 (8.2)
AIS6	2.2 ± 1.6	1.1 ± 0.4	1.2 ± 0.4	1.3 ± 0.6	0.0 ± 0.0	0.0 ± 0.0	1.0 ± 0.0	3.3 ± 1.5	0.0 ± 0.0	3.1 ± 1.6	0.0 ± 0.0	2.2 ± 1.6
AIS6 ≥ 3	53 (0.5)	1 (0.0)	0 (0.0)	0 (0.0)	0 (0.0)	0 (0.0)	0 (0.0)	47 (56.0)	0 (0.0)	4 (57.1)	0 (0.0)	1 (0.3)

*Note:* A: traffic accident; B: fall; C: crush; D: slip down; E: stab or cutting; F: machine; G: others (sexual assault, chemical burn, electric shock); H: choking; I: nonchemical burn; J: drowning; K: unknown.

Abbreviations: AIS = Abbreviated Injury Scale, AIS1 = head and neck AIS, AIS2 = facial AIS, AIS3 = chest AIS, AIS4 = intra-abdominopelvic organ AIS, AIS5 = extremity and pelvic bone AIS, AIS6 = external AIS.

^1^Data are expressed as *n* (%) unless otherwise noted.

**Table 2:** Accidental patients and self-harm patients' clinical characteristics analyzed by mechanisms.

Accidental patients	Overall^1^ (*N* = 9715)	A^1^ (*N* = 5977)	B^1^ (*N* = 1967)	C^1^ (*N* = 585)	D^1^ (*N* = 466)	E^1^ (*N* = 141)	F^1^ (*N* = 177)	G^1^ (*N* = 76)	H^1^ (*N* = 1)	I^1^ (*N* = 7)	J^1^ (*N* = 3)	K^1^ (*N* = 315)
Age (years)	53.8 ± 19.5	52.0 ± 20.4	55.8 ± 17.1	54.9 ± 17.5	64.6 ± 18.4	48.5 ± 17.9	55.8 ± 14.0	49.4 ± 17.0	74.0 ± 0.0	47.6 ± 18.8	34.0 ± 3.6	61.9 ± 16.8
Sex												
Female	2561 (26.3)	1800 (30.1)	374 (19.0)	93 (15.9)	166 (35.6)	27 (19.1)	14 (7.9)	12 (15.8)	0 (0.0)	2 (28.6)	0 (0.0)	73 (23.2)
Male	7154 (73.6)	4177 (69.9)	1593 (81.0)	492 (84.1)	300 (64.4)	114 (80.9)	163 (92.1)	64 (84.2)	1 (100.0)	5 (71.4)	3 (100.0)	242 (76.8)
ISS	13.6 ± 10.1	12.9 ± 10.2	15.5 ± 10.1	14.3 ± 8.6	14.2 ± 9.3	6.5 ± 7.3	10.9 ± 9.4	15.5 ± 15.0	9.0 ± 0.0	17.9 ± 19.5	16.3 ± 8.5	18.7 ± 9.7

**Self-harm patients**	**Overall^1^ (*N* = 307)**	**A^1^ (*N* = 14)**	**B^1^ (*N* = 126)**	**C^1^ (*N* = 2)**	**D^1^ (*N* = 1)**	**E^1^ (*N* = 150)**	**F^1^ (*N* = 0)**	**G^1^ (*N* = 0)**	**H^1^ (*N* = 8)**	**I^1^ (*N* = 4)**	**J^1^ (*N* = 2)**	**K^1^ (*N* = 0)**

Age (years)	43.6 ± 18.2	49.9 ± 11.7	36.5 ± 18.3	54.5 ± 2.1	85.0 ± 0.0	47.8 ± 17.0	0.0 ± 0.0	0.0 ± 0.0	54.9 ± 11.0	54.5 ± 8.0	32.5 ± 23.3	0.0 ± 0.0
Sex												
Female	122 (39.7)	3 (21.4)	63 (50.0)	1 (50.0)	0 (0.0)	50 (33.3)	0 (0.0)	0 (0.0)	2 (25.0)	1 (25.0)	2 (100.0)	0 (0.0)
Male	185 (60.3)	11 (78.6)	63 (50.0)	1 (50.0)	1 (100.0)	100 (66.7)	0 (0.0)	0 (0.0)	6 (75.0)	3 (75.0)	0 (0.0)	0 (0.0)
ISS	11.7 ± 11.6	10.7 ± 11.0	18.6 ± 13.2	11.5 ± 7.8	16.0 ± 0.0	5.7 ± 5.6	0.0 ± 0.0	0.0 ± 0.0	14.8 ± 11.0	19.8 ± 6.2	4.0 ± 0.0	0.0 ± 0.0

*Note:* A: traffic accident; B: fall; C: crush; D: slip down; E: stab or cutting; F: machine; G: others (sexual assault, chemical burn, electric shock); H: choking; I: nonchemical burn; J: drowning; K: unknown.

Abbreviations: AIS = Abbreviated Injury Scale, AIS1 = head and neck AIS, AIS2 = facial AIS, AIS3 = chest AIS, AIS4 = intra-abdominopelvic organ AIS, AIS5 = extremity and pelvic bone AIS, AIS6 = external AIS.

^1^Data are expressed as *n* (%) unless otherwise note.

**Table 3:** The correlation between injury mechanisms and injury sites of all-patients group.

	AIS1	AIS2	AIS3	AIS4	AIS5	AIS6	*p* value
A	2.5 ± 1.3^2,3,4,5,6^	1.4 ± 0.6^1,3,4,5^	2.6 ± 1.2^1,2,4,5,6^	2.3 ± 1.1^1,2,3,5,6^	2.0 ± 0.9^1,2,3,4,6^	1.1 ± 0.4^1,3,4,5^	< 0.0001
B	3.0 ± 1.4^2,4,5,6^	1.5 ± 0.6^1,3,4,5^	2.9 ± 1.2^2,4,5,6^	2.4 ± 0.8^1,2,3,5,6^	2.2 ± 0.9^1,2,3,4,6^	1.2 ± 0.4^1,3,4,5^	< 0.0001
C	3.2 ± 1.4^2,4,5^	1.4 ± 0.6^1,3,4,5^	2.7 ± 0.9^2,5^	2.5 ± 0.7^1,2^	2.3 ± 1.0^1,2,3^	1.3 ± 0.6	< 0.0001
D	3.6 ± 1.2^2,3,4,5^	1.4 ± 0.5^1,3,4,5^	2.9 ± 1.1^1,2,5^	2.5 ± 0.7^1,2^	2.1 ± 0.8^1,2,3^	0.0 ± 0.0	< 0.0001
E	2.0 ± 1.4^2,3^	1.3 ± 0.5^1,3,4^	2.4 ± 1.2^1,2,4,5^	1.9 ± 1.1^2,3^	1.7 ± 0.8^3^	0.0 ± 0.0	< 0.0001
F	2.9 ± 1.8^2^	1.6 ± 0.6^1,3,4,5^	2.6 ± 0.7^2^	2.5 ± 1.1^2^	2.4 ± 0.9^2^	1.0 ± 0.0	< 0.0001
G	2.5 ± 1.1	1.3 ± 0.5^6^	2.6 ± 1.0	2.2 ± 0.4	2.1 ± 0.9^6^	3.3 ± 1.5^2,5^	0.0001
H	4.0 ± 1.1	0.0 ± 0.0	2.5 ± 0.7	2.0 ± 0.0	1.5 ± 0.7	0.0 ± 0.0	0.0397
I	2.0 ± 0.0	1.0 ± 0.0	3.0 ± 1.4	4.0 ± 0.0	2.0 ± 0.0	3.1 ± 1.6	0.5079
J	3.3 ± 1.5	0.0 ± 0.0	2.0 ± 0.0	0.0 ± 0.0	0.0 ± 0.0	0.0 ± 0.0	0.2733
K	3.8 ± 1.3^2,3,4,5^	1.5 ± 0.6^1,3,4,5^	2.8 ± 1.4^1,2,5^	2.3 ± 0.8^1,2^	2.0 ± 1.1^1,2,3^	2.2 ± 1.6	< 0.0001

*Note:* A: traffic accident; B: fall; C: crush; D: slip down; E: stab or cutting; F: machine; G: others (sexual assault, chemical burn, electric shock); H: choking; I: nonchemical burn; J: drowning; K: unknown.

Abbreviations: AIS = Abbreviated Injury Scale, AIS1 = head and neck AIS, AIS2 = facial AIS, AIS3 = chest AIS, AIS4 = intra-abdominopelvic organ AIS, AIS5 = extremity and pelvic bone AIS, AIS6 = external AIS.

^1^Significant difference was observed between AIS1 and AIS2 or AIS3 or AIS4 or AIS5 or AIS6 as a result of post hoc analysis.

^2^Significant difference was observed between AIS2 and AIS1 or AIS3 or AIS4 or AIS5 or AIS6 as a result of post hoc analysis.

^3^Significant difference was observed between AIS3 and AIS1 or AIS2 or AIS4 or AIS5 or AIS6 as a result of post hoc analysis.

^4^Significant difference was observed between AIS4 and AIS1 or AIS2 or AIS3 or AIS5 or AIS6 as a result of post hoc analysis.

^5^Significant difference was observed between AIS5 and AIS1 or AIS2 or AIS3 or AIS4 or AIS6 as a result of post hoc analysis.

^6^Significant difference was observed between AIS6 and AIS1 or AIS2 or AIS3 or AIS4 or AIS5 as a result of post hoc analysis.

**Table 4:** The correlation between injury mechanisms and injury sites of accidental group and self-harm group (ISS ≥ 16).

Accidental patients	AIS1	AIS2	AIS3	AIS4	AIS5	AIS6	*p* value
A	3.2 ± 1.3^2,4,5,6^	1.6 ± 0.7^1,3,4,5^	3.0 ± 0.8^2,4,5,6^	2.7 ± 1.0^1,2,3,5^	2.4 ± 1.0^1,2,3,4^	1.5 ± 1.1^1,3^	< 0.0001
B	3.6 ± 1.2^2,3,4,5,6^	1.6 ± 0.7^1,3,4,5^	3.0 ± 1.0^1,2,4,5,6^	2.4 ± 0.7^1,2,3^	2.4 ± 0.9^1,2,3^	1.0 ± 0.0^1,3^	< 0.0001
C	4.0 ± 1.3^2,3,4,5^	1.6 ± 0.7^1,3,4,5^	3.0 ± 0.9^1,2^	2.7 ± 0.8^1,2^	2.8 ± 1.1^1,2^	1.5 ± 0.7	< 0.0001
D	4.4 ± 0.7^2,3,4,5^	1.4 ± 0.5^1,3,4^	3.4 ± 1.3^1,2,4^	2.9 ± 0.8^1,2^	2.1 ± 1.1^1,3^	0.0 ± 0.0	< 0.0001
E	3.9 ± 1.0	2.0 ± 0.8	3.6 ± 1.1	3.0 ± 0.8	2.6 ± 1.5	0.0 ± 0.0	0.0598
F	3.6 ± 1.8^2^	1.6 ± 0.7^1,3,5^	2.9 ± 0.4^2^	2.9 ± 1.1	3.0 ± 1.1^2^	0.0 ± 0.0	0.0065
G	3.0 ± 1.3	1.3 ± 0.5^6^	3.0 ± 1.0	2.3 ± 0.6	2.2 ± 1.1^6^	4.6 ± 0.8^2,5^	< 0.0001
H	.	.	.	.	.	.	.
I	2.0 ± 0.0	0.0 ± 0.0	4.0 ± 0.0	4.0 ± 0.0	2.0 ± 0.0	4.7 ± 0.6	0.1832
J	4.5 ± 0.7	0.0 ± 0.0	0.0 ± 0.0	0.0 ± 0.0	0.0 ± 0.0	0.0 ± 0.0	.
K	4.2 ± 1.0^2,3,4,5^	1.5 ± 0.6^1,3^	2.9 ± 1.0^1,2^	2.4 ± 0.8^1^	2.2 ± 1.3^1^	3.0 ± 2.8	< 0.0001

**Self-harm patients**	**AIS1**	**AIS2**	**AIS3**	**AIS4**	**AIS5**	**AIS6**	***p* value**

A	3.3 ± 1.5	1.5 ± 0.7	3.0 ± 0.0	0.0 ± 0.0	2.3 ± 0.6	0.0 ± 0.0	0.1544
B	3.5 ± 2.3^2^	1.8 ± 0.9^1,3,4,5^	3.0 ± 1.4^2^	2.7 ± 0.8^2^	3.0 ± 1.1^2^	1.0 ± 0.0	< 0.0001
C	2.0 ± 0.0	0.0 ± 0.0	2.0 ± 0.0	3.0 ± 0.0	0.0 ± 0.0	0.0 ± 0.0	0.3679
D	4.0 ± 0.0	0.0 ± 0.0	0.0 ± 0.0	0.0 ± 0.0	0.0 ± 0.0	0.0 ± 0.0	.
E	3.2 ± 1.5	0.0 ± 0.0	3.5 ± 1.0	3.4 ± 1.0	2.0 ± 1.4	0.0 ± 0.0	0.5353
F	.	.	.	.	.	.	.
G	.	.	.	.	.	.	.
H	4.2 ± 1.1	0.0 ± 0.0	2.5 ± 0.7	2.0 ± 0.0	2.0 ± 0.0	0.0 ± 0.0	0.1253
I	0.0 ± 0.0	0.0 ± 0.0	0.0 ± 0.0	0.0 ± 0.0	0.0 ± 0.0	4.7 ± 0.6	.
J	.	.	.	.	.	.	.
K	.	.	.	.	.	.	.

*Note:* A: traffic accident; B: fall; C: crush; D: slip down; E: stab or cutting; F: machine; G: others (sexual assault, chemical burn, electric shock); H: choking; I: nonchemical burn; J: drowning; K: unknown.

Abbreviations: AIS = Abbreviated Injury Scale, AIS1 = head and neck AIS, AIS2 = facial AIS, AIS3 = chest AIS, AIS4 = intra-abdominopelvic organ AIS, AIS5 = extremity and pelvic bone AIS, AIS6 = external AIS.

^1^Significant difference was observed between AIS1 and AIS2 or AIS3 or AIS4 or AIS5 or AIS6 as a result of post hoc analysis.

^2^Significant difference was observed between AIS2 and AIS1 or AIS3 or AIS4 or AIS5 or AIS6 as a result of post hoc analysis.

^3^Significant difference was observed between AIS3 and AIS1 or AIS2 or AIS4 or AIS5 or AIS6 as a result of post hoc analysis.

^4^Significant difference was observed between AIS4 and AIS1 or AIS2 or AIS3 or AIS5 or AIS6 as a result of post hoc analysis.

^5^Significant difference was observed between AIS5 and AIS1 or AIS2 or AIS3 or AIS4 or AIS6 as a result of post hoc analysis.

^6^Significant difference was observed between AIS6 and AIS1 or AIS2 or AIS3 or AIS4 or AIS5 as a result of post hoc analysis.

**Table 5:** Number of each age groups in self-harm group by mechanisms.

Age (years)	Overall^1^ (*N* = 307)	A^1^ (*N* = 14)	B^1^ (*N* = 126)	C^1^ (*N* = 2)	D^1^ (*N* = 1)	E^1^ (*N* = 150)	F^1^ (*N* = 0)	G^1^ (*N* = 0)	H^1^ (*N* = 8)	I^1^ (*N* = 4)	J^1^ (*N* = 2)	K^1^ (*N* = 0)
0∼9	1 (0.3)	0 (0.0)	1 (0.8)	0 (0.0)	0 (0.0)	0 (0.0)	0 (0.0)	0 (0.0)	0 (0.0)	0 (0.0)	0 (0.0)	0 (0.0)
10∼19	33 (10.8)	0 (0.0)	26 (20.6)	0 (0.0)	0 (0.0)	6 (4.0)	0 (0.0)	0 (0.0)	0 (0.0)	0 (0.0)	1 (50.0)	0 (0.0)
20∼29	48 (15.6)	1 (7.1)	29 (23.0)	0 (0.0)	0 (0.0)	18 (12.0)	0 (0.0)	0 (0.0)	0 (0.0)	0 (0.0)	0 (0.0)	0 (0.0)
30∼39	46 (15.0)	1 (7.1)	23 (18.3)	0 (0.0)	0 (0.0)	22 (14.7)	0 (0.0)	0 (0.0)	0 (0.0)	0 (0.0)	0 (0.0)	0 (0.0)
40∼49	67 (21.8)	6 (42.9)	17 (13.5)	0 (0.0)	0 (0.0)	39 (26.0)	0 (0.0)	0 (0.0)	3 (37.5)	1 (25.0)	1 (50.0)	0 (0.0)
50∼59	54 (17.6)	4 (28.6)	15 (11.9)	2 (100.0)	0 (0.0)	30 (20.0)	0 (0.0)	0 (0.0)	2 (25.0)	1 (25.0)	0 (0.0)	0 (0.0)
60∼69	28 (9.1)	1 (7.1)	8 (6.4)	0 (0.0)	0 (0.0)	15 (10.0)	0 (0.0)	0 (0.0)	2 (25.0)	2 (50.0)	0 (0.0)	0 (0.0)
70∼79	21 (6.8)	1 (7.1)	5 (4.0)	0 (0.0)	0 (0.0)	14 (9.3)	0 (0.0)	0 (0.0)	1 (12.5)	0 (0.0)	0 (0.0)	0 (0.0)
80∼89	7 (2.3)	0 (0.0)	1 (0.8)	0 (0.0)	1 (100.0)	5 (3.3)	0 (0.0)	0 (0.0)	0 (0.0)	0 (0.0)	0 (0.0)	0 (0.0)
90∼	2 (0.7)	0 (0.0)	1 (0.8)	0 (0.0)	0 (0.0)	1 (0.7)	0 (0.0)	0 (0.0)	0 (0.0)	0 (0.0)	0 (0.0)	0 (0.0)

*Note:* A: traffic accident; B: fall; C: crush; D: slip down; E: stab or cutting; F: machine; G: others (sexual assault, chemical burn, electric shock); H: choking; I: nonchemical burn; J: drowning; K: unknown.

Abbreviations: AIS = Abbreviated Injury Scale, AIS1 = head and neck AIS, AIS2 = facial AIS, AIS3 = chest AIS, AIS4 = intra-abdominopelvic organ AIS, AIS5 = extremity and pelvic bone AIS, AIS6 = external AIS.

^1^Data are expressed as *n* (%) unless otherwise noted.

**Table 6:** The correlation between injury sites and intentionality of fall-down injury mechanism.

	A	B	*p* value	Effect size *r*^1^
AIS1	3.0 ± 1.4 (*N* = 1161)	2.8 ± 2.0 (*N* = 52)	0.0054	−0.08
AIS2	1.5 ± 0.6 (*N* = 584)	1.6 ± 0.8 (*N* = 39)	0.7827	0.01
AIS3	2.8 ± 1.1 (*N* = 915)	3.1 ± 1.9 (*N* = 82)	0.7225	−0.01
AIS4	2.3 ± 0.7 (*N* = 612)	2.7 ± 1.3 (*N* = 74)	0.0194	0.09
AIS5	2.2 ± 0.8 (*N* = 1043)	2.7 ± 1.1 (*N* = 99)	< 0.0001	0.14
AIS6	1.2 ± 0.4 (*N* = 10)	1.0 ± 0.0 (*N* = 2)	0.6188	−0.14

*Note:* A: accidental patients with fall injury mechanism; B: self-harm patients with fall injury mechanism.

Abbreviations: AIS = Abbreviated Injury Scale, AIS1 = head and neck AIS, AIS2 = facial AIS, AIS3 = chest AIS, AIS4 = intra-abdominopelvic organ AIS, AIS5 = extremity and pelvic bone AIS, AIS6 = external AIS.

^1^Effect size *r* was calculated by formula $r=\left(Z/\sqrt{N}\right)$.

## Data Availability

The data that support the findings of this study are available from the corresponding author upon reasonable request.
